# Metabolomics and sensory evaluation combined analysis reveal the effect of processing methods and different forms on the flavor of rose herbal tea

**DOI:** 10.1038/s41538-025-00387-x

**Published:** 2025-03-29

**Authors:** Yue Zhao, Qunhua Peng, Lu Chang, Zhe Wang, Zhi Lin, Jiang Shi, Chunwang Dong

**Affiliations:** 1 Tea Research Institute, Shandong Academy of Agricultural Sciences, Jinan, China; 2 Key Laboratory of Tea Biology and Resource Utilization of Ministry of Agriculture, Tea Research Institute, Chinese Academy of Agricultural Sciences, Hangzhou, China; 3 Shandong Huamei Biotechnology Co., Ltd, Jinan, China

**Keywords:** Metabolomics, Nutrition

## Abstract

Pingyin rose herbal tea is favored in the market for its unique quality and health benefits. Thus, researchers have explored a range of different rose products. This study evaluated the effect of processing methods and different forms on the flavor of rose herbal tea combined with sensory evaluation and metabolite profiles. Sensory evaluation showed that low-temperature drying (LTD) roses have a distinct floral and sweet aroma, while vacuum freeze drying (VFD) roses exhibit a fruity and woody aroma. Metabolomics analysis indicated that each type of rose herbal tea has its characteristic accumulation of non-volatile compounds and volatile organic compounds (VOCs). The VFD rose corollas had the highest contents of AAs, OAs, SSs, flavonoids, and VOCs. Furthermore, correlation analysis revealed key nonvolatile and volatile compounds related to aroma and taste. This study provides a scientific foundation for future investigations on the processing and quality improvement of rose herbal tea.

## Introduction

Pingyin rose (*Rosa rugosa* cv. Plena) is known for its layered petals, bright color, and rich aroma, and is therefore a high-quality raw material for making rose tea^1^. Rose herbal tea is also rich in various nutrients and active substances, including amino acids, organic acids, soluble sugars (SSs), and flavonoids, with various effects such as beauty and beauty, regulating Qi and blood, and soothing emotions, making it an ideal beverage for modern people to pursue a healthy life^2–4^. Therefore, these unique edible rose product resources have attracted much attention on the market and are considered to be medicinal and edible homologous plants, demonstrating good commercial production value.

The quality and nutrient retention of rose tea are greatly influenced by the drying process. The two most popular drying methods are vacuum freeze-drying (VFD) and low-temperature drying (LTD). LTD, also known as hot air drying (HAD), is used extensively for drying agricultural and food products due to its reduced investment cost and better drying condition control^5^. The HAD rose tea sample was closest to neutral in pH and had the highest VC level^1^. The VFD roses had a obvious bright red color, low shrinkage, good plasma membrane permeability, and complete tissue cells. VFD can effectively prevent odor reduction/destruction and maintain the natural scent of roses^6^. Better drying efficiency is demonstrated by HAD, but according to product quality, VFD has a greater benefit^7^. Furthermore, the content and composition of metabolites in roses also fluctuate at different stages of flower growth, which affects quality and flavor of rose tea^8^. Therefore, it is crucial to investigate the flavor of rose herbal tea in different states commonly found in the market.

The flavor of rose herbal tea comes from its non-volatile compounds and aroma components (VOCs, volatile organic compounds)^9^. The main VOCs of rose tea are alcohols, esters, aldehydes, and terpenes, which are produced by four main precursors, namely carotenoids, amino acids, glycosides, and lipids, through reactions such as oxidation, polymerization, condensation, and group transfer^1,10^. The identified alcohol compounds emphasized the floral and fruity aromas^11^. Rose samples frequently contain unsaturated aldehydes, and in comparison to saturated aldehydes, have a more potent and appealing flavor^12^.

The non-volatile compounds mainly affect its taste. Present study largely focuses on the changes in the content and biological activity of non-volatile compounds in rose herbal tea after different drying methods. Phenol content is positively correlated with antioxidant capacity, and plant chemicals are most prominent in unfolded petals^13^. After drying, the composition and biological activity of roses increase, especially the content of anthocyanins^14^. At different temperatures, the total phenolic content of the IRPSFD samples is slightly greater^15^. However, the above research is not comprehensive enough. Research on the specific components of nonvolatile chemicals, particularly the SSs, amino acids, and catechins found in rose tea, and their synergistic effects with flavor is comparatively lacking. The key non-volatile compounds contained in rose tea and their contribution to the flavor of rose tea are still unknown.

Therefore, different forms of roses grown under the same environmental conditions were used as materials to conduct sensory and quality analyses of rose products under different drying methods (LTD and VFD). This study aimed to thoroughly examine the accumulation profile of the nonvolatile components, such as flavonoids, particularly anthocyanins, SSs, amino acids, and organic acids, and VOCs in various forms of rose tea under LTD and VFD processes, as well as their synergistic effects on flavor sensory quality. In terms of flavor orientation, an analysis of popular rose tea products on the market was conducted with the goal of increasing economic value and offering helpful information for large-scale industrial operations.

## Results

### Sensory evaluation of rose herbal tea

**Fig. 1:**
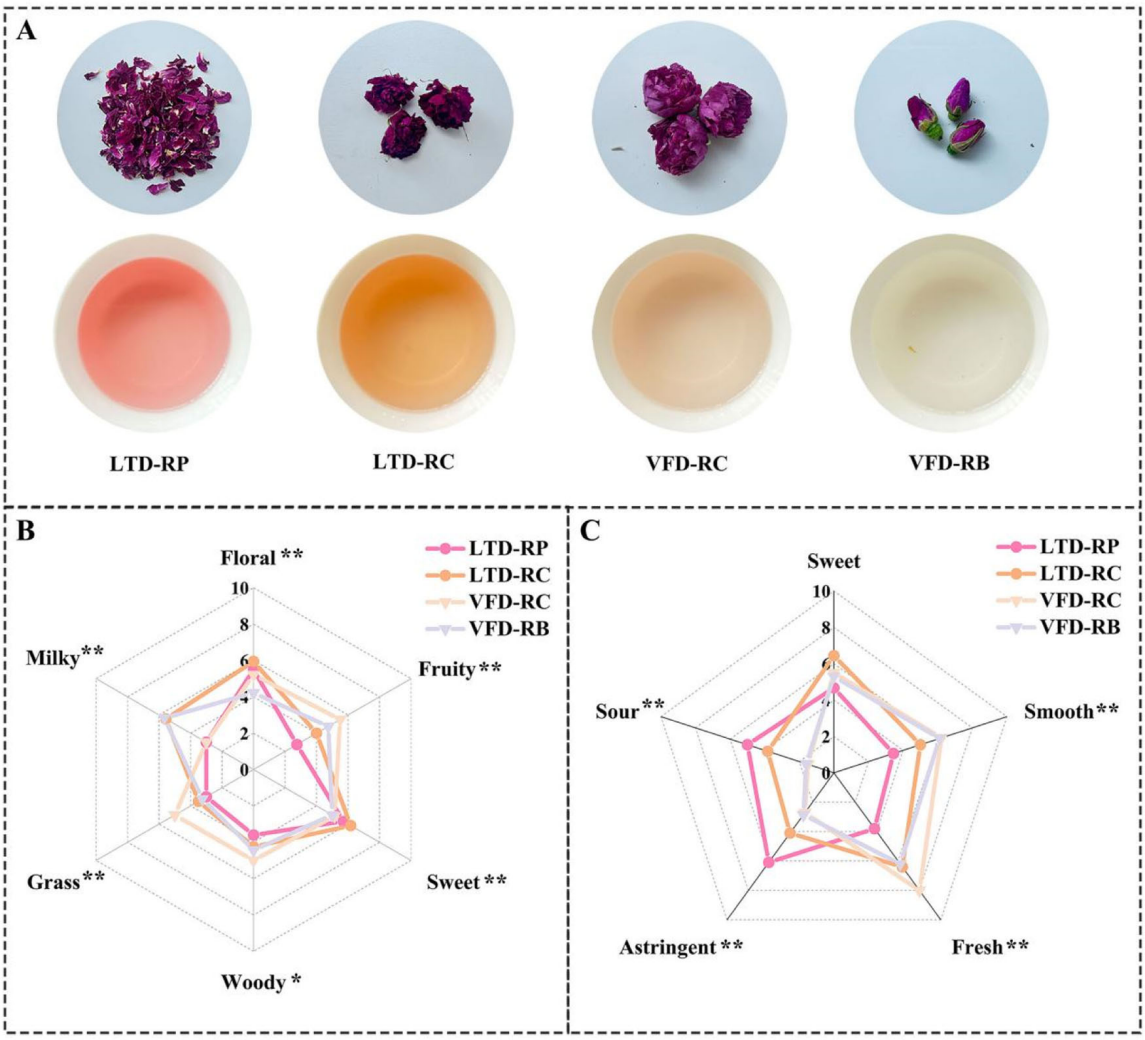
Sensory quality analysis of four rose herbal teas. **A** Representative appearance and infusion of four rose herbal teas. **B** Radar map of the quantitative descriptive analysis of aroma scores. **C** Radar map of the quantitative descriptive analysis of taste scores. LTD-RP Low-temperature drying rose petal, LTD-RC Low-temperature drying rose corolla, VFD-RC vacuum freeze-drying rose corolla, VFD-RB vacuum freeze-drying rose bud. *p < 0.05, **p < 0.01.

The sensory quality of rose herbal tea was evaluated using traditional sensory evaluations. The color of LTD roses and their aqueous tea infusions were darker, which results from ascorbic acid browning, the Maillard reaction, and the caramel reaction^16^. VFD roses have the freshest red color, while the color of rose tea infusions is lighter (Fig. 1A). The color intensity of rose petals and their tea infusion depends on the type and content of metabolic compounds contained. Vacuum and low temperature environment suppresses the oxidation reactions and enzymatic browning of roses, thereby better maintaining the original color of the sample^17^.

Similarly, those rose herbal teas have distinct sensory characteristics (Supplementary Data 1). The results demonstrated that LTD rose tea has higher floral and sweet intensity than other aromas, while VFD rose tea has higher intensity of fruity and woody aromas than other aromas. In addition, the RCs of rose tea (LTD-RC and VFD-RC) had distinct milky aromas in aroma (Fig. 1B). The taste characteristics of rose tea vary greatly among different forms and processes. The RCs (LTD-RC and VFD-RC) exhibited distinct sweetness, especially LTD-RC. LTD-RP exhibited distinct flavor characteristics, including strong sourness and astringency, together with low-intensity freshness, smoothness, and sweetness. This may be due to the fact that polyphenolic compounds in LTD-RP, especially anthocyanins, are more easily dissolved in rose tea infusion^15^. Furthermore, the drying process of the VFD results in a smoother and fresher taste of the rose infusion (Fig. 1C). In summary, different forms and dried processed rose teas have specific flavor properties, it is crucial to investigate the flavor characteristics components of rose tea.

### AAs differential accumulation in different forms of rose herbal tea using LTD and VFD

Amino acids and their derivatives (AAs) are the primary participants in the flavor of rose tea. They have unique sensory characteristics and physiological functions, and play crucial roles in aroma, taste, and even health benefits^18^. In this study, there were 78 kinds of AAs were identified. In four rose herbal teas, γ-aminobutyric acid was the most prominent amino acid, followed by L-glutamic acid, L-asparagine anhydrous, L-glutamine, and N-acetylneuraminic acid (Supplementary Data 2a).

**Fig. 2:**
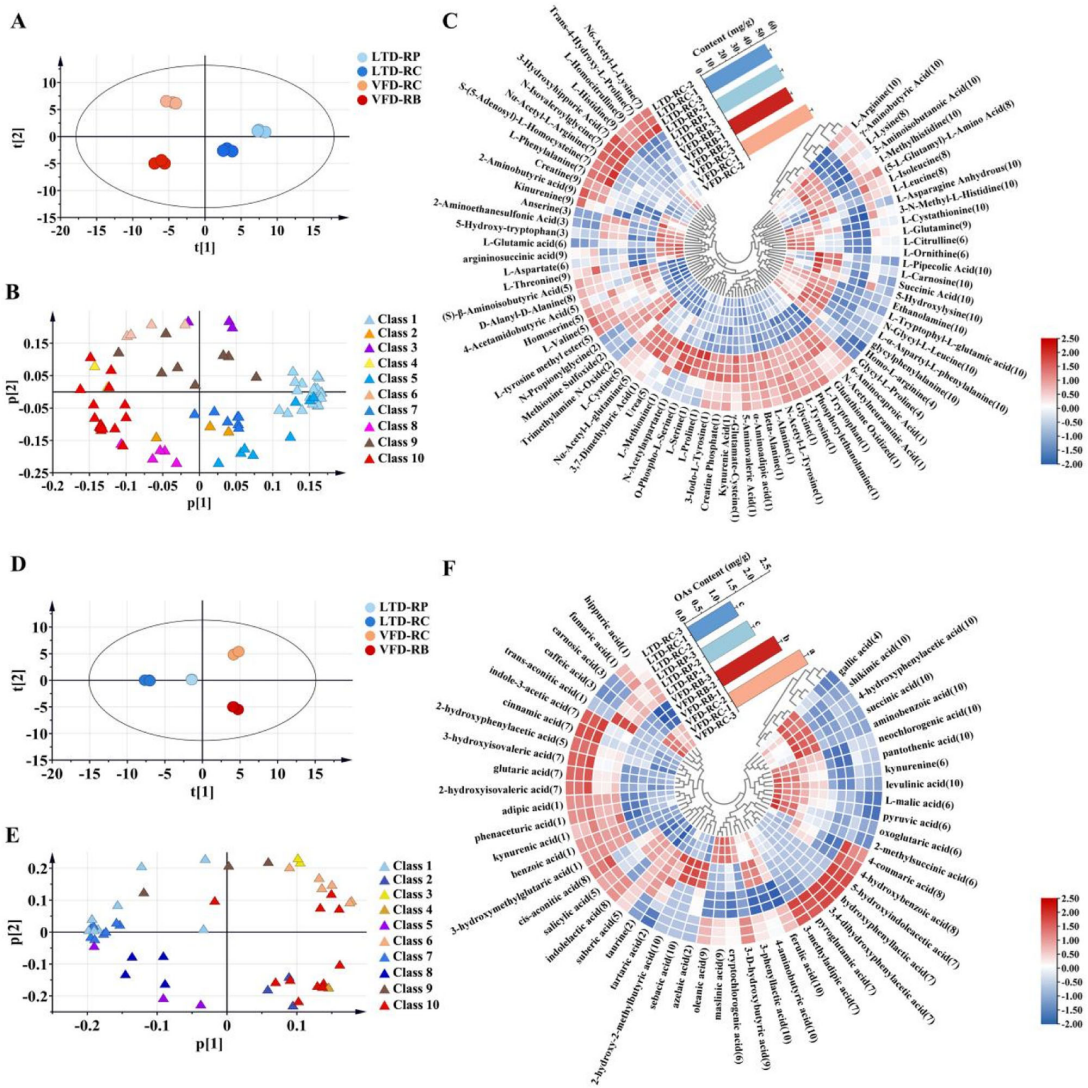
Amino acids (AAs) and organic acids (OAs) profiles of four rose samples and drying techniques. Scatter plot of the principal component analysis (PCA) scores of AAs (**A**) and OAs (**D**). Different colored boxes indicate different types: light blue, LTD-RP; dark blue, LTD-RC; orange red, VFD-RC; red, VFD-RB. Loading scatter plot of the PCA based on p1 versus p2 for AAs (**B**) and OAs (**E**). The different colored boxes indicate different classes. Hierarchical cluster analysis (HCA) and bar charts of AAs (**C**) and OAs (**F**) accumulation patterns among the four types of rose herbal teas. The contents of four rose herbal teas. Significant differences among various rose samples were calculated using one-way ANOVA (LSD test) in IBM SPSS. The different letters indicated significant differences.

To examine the variations in the accumulation profile of AAs in the four types of rose tea, principal component analysis (PCA) was performed. The scatter plot of the PCA scores showed that the four types of rose tea were divided into three categorie: the LTD samples were grouped together, and the VFD-RB and VFD-RC samples were separated (Fig. 2A). This indicates that the degree of flower development and the drying procedure have an impact on the amount of amino acids that accumulate in rose tea. Trend clustering analysis (TCA) revealed all the AAs in the four rose herbal tea could clustered into 10 classes (Supplementary Fig. 1A). The AAs in classes 1, 5, and 7 were the key AAs affecting the distribution of the LTD samples, while the AAs in classes 4, 6, 8, and 10 were the key AAs affecting the grouping of the VFD samples (Fig. 2B).

There are differences in the 78 amino acid accumulation patterns among the four varieties of rose tea, although the total amount of amino acids has no significant difference (Fig. 2C). Among the four rose herbal teas, VFD-RC had the most amino acids (51.97 mg/g), followed by LTD-RC (48.69 mg/g), LTD-RP (48.53 mg/g), and VFD-RB (46.05 mg/g). The results of hierarchical clustering analysis (HCA) showed that the AAs in class 1 (22 AAs), such as N-acetylneuraminic acid, L-tryptophan, glycine, and L-alanine, mainly accumulate in the LTD samples. L-proline and L-serine were more abundant in LTD-RP, while L-phenylalanine and Nα-acetyl-L-arginine were more abundant in LTD-RC. Moreover, rose tea processed by VFD accumulated more L-tryptophyl-L-glutamic acid, ethanolamine, and L-α-aspartyl-L-phenylalanine. In general, all those four types of rose herbal tea have accumulated characteristic amino acids, although there was no significant difference in the total amount of amino acids between them.

### Differential accumulation of OAs in different forms of rose herbal tea using LTD and VFD

Sour taste is usually tasted in rose tea brewing and has a negative impact on sensory quality. Acidic metabolites can affect the flavor of tea, and organic acids (OAs) are considered one of the main factors^19^. The organic acid composition of different types of tea varies, and the primary OAs in black tea are citric acid and quinic acid^20^. The most prominent organic acids detected in rose herbal tea were L-malic acid, maslinic acid, tartaric acid, and cis-aconitic acid (Supplementary Data 2b). Among them, L-malic acid was dominant. Malic acid improves oral health in patients with dry mouth syndrome^21^.

To explore the effects of maturity and drying methods of rose tea on the accumulation of OAs, PCA was conducted. The four finished rose teas were clustered separately, and the LTD samples were clustered together, and separated from VDD-RB and VFD-RC. This indicates a significant impact of flower maturity and drying method on organic acid content (Fig. 2D). Based on TCA, the compound loading graph showed that these 52 OAs could be divided into 10 classes (Fig. 2E, Supplementary Fig. 1B). The OAs in classes 3, 4, 6, and 10 were the key OAs that mainly accumulated in the VFD samples, while the OAs in classes 1, 5, 7, and 8 were the key OAs that mainly accumulated in the LTD samples.

The overall quantity of OAs did not significantly differ between the LTD samples, but it did significantly differ between the VFD samples and between the VFD and LTD samples. The OAs content of VFD-RC was 2.10 mg/g, followed by those of VFD-RB, LTD-RP, and LTD-RC (Fig. 2F). The differential accumulation of several organic acids in rose tea was further examined using HCA. The 6 OAs in Group 6, including L-malic acid, oxoglutaric acid, pyruvic acid, cryptochlorogenic acid, 2-methylsuccinic acid, and kynurenine, were more abundant in VFD-RC than in the other groups. In VFD-RP, gallic acid, shikimic acid, succinic acid, aminobenzoic acid, 4-hydroxyphenylacetic acid, 2-hydroxy-2-methylbutyric acid, azelaic acid, sebacic acid, and tartaric acid accumulate more. Specifically, azelaic acid, sebacic acid, and 2-hydroxy-2-methylbutyric acid accumulate at high levels in VFD-RP. Gallic acid (GA) and succinic acid (SA) were discovered to strengthened the umami taste^22^. Further explanation showed that flower maturity and drying methods significantly affect the accumulation of OAs.

### Differential accumulation of SSs in different forms of rose herbal tea using LTD and VFD

**Fig. 3:**
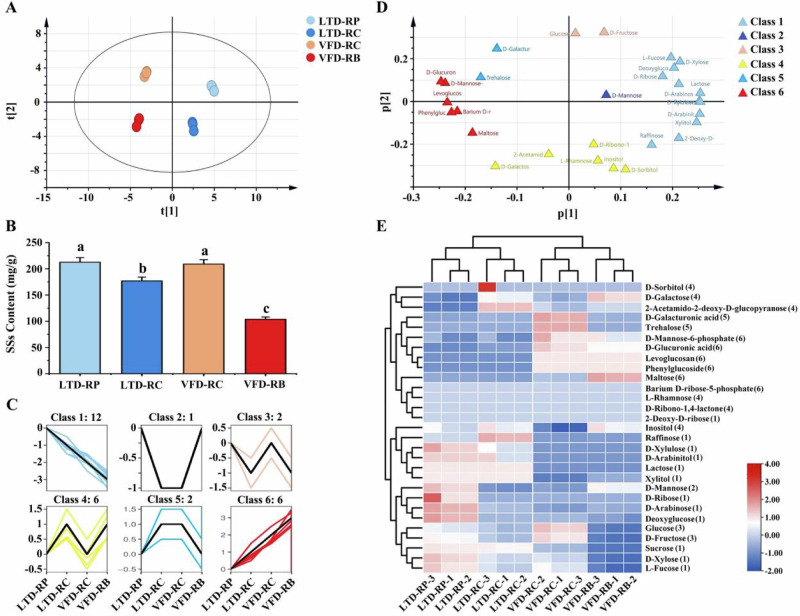
Soluble sugars profiles in four rose samples and drying techniques. **A** Scatter plot of the principal component analysis (PCA) score based on t1 versus t2. Different colored boxes indicate different types: light blue, LTD-RP; dark blue, LTD-RC; orange red, VFD-RC; red, VFD-RB. **B** The contents of soluble sugars in four rose herbal teas. Significant differences among various rose samples were calculated using one-way ANOVA (LSD test) in IBM SPSS. The different letters indicate significant differences. **C** Trend clustering analysis showing differences in soluble sugar accumulation patterns among the four types of rose herbal teas. **D** PCA loading scatter plot based on p1 versus p2. The different colored boxes indicate the different soluble sugar classes. **E** Hierarchical cluster analysis (HCA) of soluble sugars in four rose herbal teas.

SSs can conduce to the formation of aroma and sweetness in rose herbal tea^23^. In this study, there were 29 kinds of SSs identified in the four rose herbal teas. The most abundant soluble sugar among the four types of rose tea was glucose, which reached 95.61 mg/g in VFD-RC, followed by fructose and sucrose, respectively (Supplementary Data 2c). The PCA score plots indicated that the four rose teas could be well distinguished based on these 29 SSs (Fig. 3A). The total amounts of these SSs in LTD-RP and VFD-RC were relatively high, but the difference was not significant. The SSs content in the VFD-RB was the lowest (Fig. 3B). This indicates that the content of SSs in rose tea is influenced mainly by the maturity of the flowers.

To better distinguish the types of SSs that accumulated in the four types of rose tea, TCA assays were conducted on the SSs mentioned above. The TCA results indicated that SSs can be divided into 6 classes based on differences in accumulation among the four types of rose tea (Fig. 3C). The loading diagram and HCA showed that 12 SSs in class 1, mainly sucrose, raffinose, and D-arabinose, were highly accumulated in LTD rose teas. The 6 SSs in class 6, mainly maltose, D-mannose-6-phosphate, and D-glucuronic acid, were highly accumulated in rose tea processed by VFD. In addition, the content of the 6 SSs in class 4 did not differ significantly among the four types of rose tea, especially L-rhamnose, D-sorbitol, and D-ribono-1,4-lactone (Fig. 3D, E). This indicates that compared to the drying methods, the open state of roses have more effects on the soluble sugar type and content of rose tea.

### Flavonoids differential accumulation in different forms of rose herbal tea using LTD and VFD

Phenolic compounds such as flavonoids and anthocyanins give roses strong antioxidant activity^24^. In this study, 69 kinds of flavonoids with contents greater than 1 μg/g were detected in four rose herbal teas, including 46 flavones and flavonols, 3 flavanols, 2 procyanidins, and 18 anthocyanins (Supplementary Data 2d). The 18 main anthocyanins were divided into 5 categories: petunidin (1), delphinidin (3), pelargonidin (2), cyanidin (6), and peonidin (6). Among the rose samples, the flavonoids content was highest in the VFD-RC (11.68 mg/g) and lowest in the LTD-RP (8.67 mg/g). During the fully open flowering period, the content of flavonoids increased^25^. This suggested that the accumulation of flavonoids was influenced by the flowering period in rose tea.

**Fig. 4:**
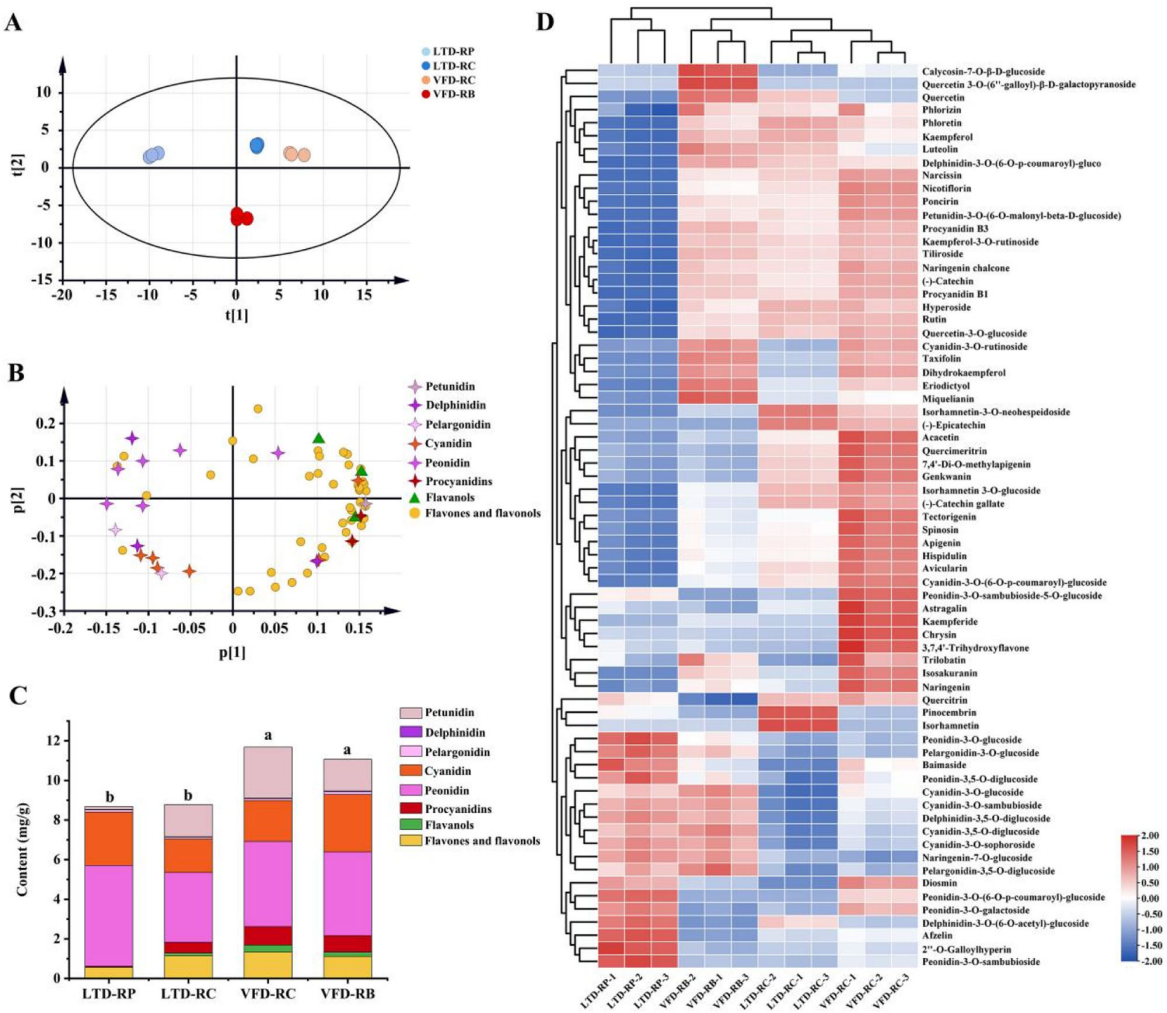
Flavonoids profiles of the four rose samples and drying techniques. **A** Scatter plot of the principal component analysis (PCA) score based on t1 versus t2. Different colored boxes indicate different types: light blue, LTD-RP; dark blue, LTD-RC; orange red, VFD-RC; red, VFD-RB. **B** PCA loading scatter plot based on p1 versus p2. Green triangles represent flavanols; yellow circles represent flavones and flavonols; Different colored 4-point stars represent different anthocyanins (petunidin, delphinidin, pelargonidin, cyanidin, and peonidin) and procyanidins. **C** The classification content of flavonoids in the four rose samples. The different colored boxes indicate the different types. **D** Hierarchical cluster analysis (HCA) of flavonoids in four rose herbal teas.

However, the scatter plot of the PCA scores revealed that it is difficult to distinguish between the four rose herbal tea (Fig. 4A). The compound loading diagram showed that the most diverse compounds in rose tea are flavonoids and flavonol glycosides (Fig. 4B). There was no appreciable difference in the total content of flavonoids amongst rose samples handled with the same drying technique, while the total flavonoids content of rose herbal tea processed by LTD and those by VFD differed significantly (Fig. 4C). Furthermore, VFD roses had a greater overall flavonoid content than LTD roses. Flavonoids in LTD samples may degrade as a result of prolonged drying and high temperatures^24^. This indicated that drying techniques have a greater impact on the flavonoid accumulation in rose herbal tea.

Anthocyanins are the main colorants in rose petals. The content of anthocyanins in LTD-RP was the highest, mainly cyanidin and peonidin, while the contents of petunidin, delphinidin, and pelargonidin were relatively low. Furthermore, LTD-RP contained the lowest amounts of procyanidins, flavanols, flavones, and flavonols (Fig. 4C). LTD-RP does not contain stamens, but it is precisely the stamens that contain more flavones and flavonols, which have a high antioxidant capacity^26^.

HCA was used to comprehensively analyze various types of flavonoids in rose tea (Fig. 4D). The most abundant anthocyanins detected in rose herbal tea were peonidin-3,5-O-diglucoside (Pn3G5G), and cyanidin-3,5-O-diglucoside (Cn3G5G). This determines the color tone of rose petals^27^. VFD-RC had the highest content of Cn3G5G and Pn3G5G, resulting in a color consistent with that of fresh flowers. The accumulation of anthocyanins in LTD-RP is relatively high, except for acylated anthocyanin glycosides and cyanidin-3-O-rutinoside. The contents of delphinidin-3,5-O-diglucoside, Pn3G5G, cyanidin-3-O-sambubioside, and cyanidin-3-O-glucoside, were generally lower in the RC stage, especially in the LTD-RC sample. Flavonoid glycosides in rose teas were mainly derivatives of glycosylated, acylated, and methylated quercetin and kaempferol. The VFD rose samples contained more catechins and anthocyanin B3. More quercetin and catechins are present in rose buds^13^. Based on the above results, according to the types and contents of flavonoids, the rose samples from the LTD and VFD groups did not aggregate well.

Therefore, the total accumulation of flavonoids in rose tea is related to the drying method, while the kinds of metabolites and their contents are related to the developmental stage of the flowers.

### VOCs profiles of different forms of rose herbal tea

The flavor of rose herbal tea is significantly influenced by aroma, which is the outcome of multiple volatile components working together. In recent years, the relative odor activity value (rOAV) has been increasingly applied by scholars to identify key flavor components in various types of food and tea^28^. Generally, an rOAV ≥ 1 signifies that the compound directly assists contribution to the flavor of the rose herbal tea. This section explores the differences in VOCs in rose corollas, buds, and petals under LTD and VFD processes.

**Fig. 5:**
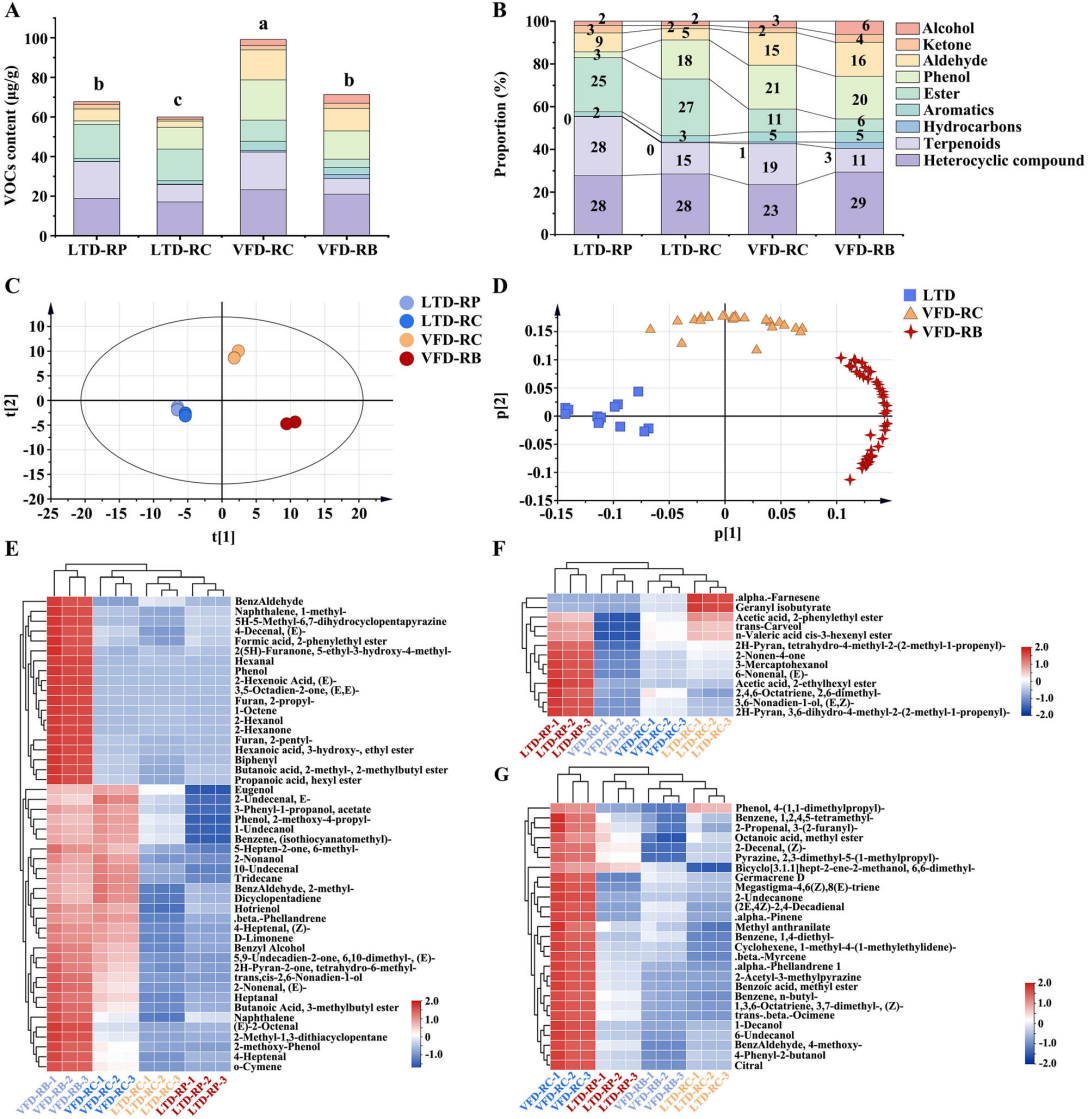
Analysis of key volatile organic compounds (VOCs) in four rose samples through GC-MS and rOAVs (rOAV ≥ 1). The classification content (**A**) and proportion (**B**) of 9 types of VOCs in four rose samples. **C** Scatter plot of the principal component analysis (PCA) score based on 88 key VOCs. The different colored circles indicate different samples: light blue, LTD-RP; dark blue, LTD-RC; orange red, VFD-RC; and red, VFD-RB. **D** PCA loading scatter plot based on p1 versus p2. Triangles of different colors represent different classes. Hierarchical cluster analysis (HCA) of VFD-RB (**E**), LTD (**F**), and VFD-RC (**G**) types VOCs in four rose herbal teas.

A total of 88 aromatic active substances (rOAV ≥ 1) were identified in the LTD-RP, LTD-RC, VFD-RC, and VFD-RB samples through GC-MS and rOAVs analysis. These substances can be divided into 9 categories, namely 11 alcohols, 6 ketones, 15 aldehydes, 4 phenols, 13 esters, 7 aromatics, 4 hydrocarbons, 17 terpenoids, and 9 heterocyclic compounds (Supplementary Data 3a). The contents of VOCs were greatest in the VFD-RC (99.18 μg/g), and lowest in the LTD-RC (59.99 μg/g) (Fig. 5A). According to recent studies, alcohol is the primary aroma component of roses^6,29^. However, our research revealed that among the 88 key VOCs, heterocyclic compounds accounted for 23–29% (9 categories) of the total VOCs the four types of rose tea. LTD samples contain more esters, while VFD samples contain more aldehydes (Fig. 5B). Notably, 3-(2-furanyl)-2-propenal, eugenol, (Z)-2-decenal, n-valeric acid cis-3-hexenyl ester, acetic acid, 2-phenylethyl ester, and trans-carveol were the abundant VOCs in Pingyin rose samples^6^.

The accumulation patterns of 88 VOCs in the four types of rose tea were examined using PCA. The scatter plot of the PCA scores showed that the four samples were dispersed into three groups, among which the LTD samples could be clearly separated from the VFD-RC and VFD-RB samples (Fig. 5C). The loading scatter plot showed that VOCs are dispersed in three different regions. Combined with the PCA score scatter plot, these three groups of VOCs were named as the LTD type, VFD-RC type, and VFD-RB type, respectively (Fig. 5D; Supplementary Data 3b).

HCA revealed the characteristic accumulation of VOCs in different rose herbal teas (Fig. 5E–G). There are 48 VOCs in the VFD-RB type, among which 19 are characteristic VOCs of the VFD-RB, including benzaldehyde, 1-methyl-naphthalene, (E)-4-decenal, 2-pentyl-furan, 1-octene, hexanal, biphenyl, and (E, E)-3,5-octadien-2-one. The remaining 29 VOCs, including eugenol, (isothiocyanatomethyl)-benzene, (Z)-4-heptenal, o-cymene, and (E)-2-nonenal are characteristic accumulated VOCs of VFD processing methods (Fig. 5E). There are 13 VOCs in the LTD type, acetic acid, 2-phenylethyl ester, trans-carveol, and n-valeric acid cis-3-hexenyl ester accumulate more in LTD processed rose tea. Among them, α-Farnesene and geranyl isobutyrate are characteristic accumulation of LTD-RC (Fig. 5F). VFD-RC type conteins 27 VOCs, such as 2-undecanone, methyl benzoate, n-butylbenzene, and 4-phenyl-2-butanol (Fig. 5G). The differences in VOCs among the different drying processes can be attributed to differences in drying mechanisms and dehydration behaviors.

### Screening of characteristic flavor metabolites of rose tea

To further identify the characteristic flavor metabolites of rose tea, partial least squares discriminant analysis (PLS-DA) was conducted on VOCs (Supplementary Fig. 2) and non-volatile compounds (Supplementary Fig. 3). The differences of volatile compounds between the LTD-RC and LTD-RP samples were not clear, while the differences between the LTD samples and the VFD-RC and VFD-RB samples were significant (Fig. 5C, D; Supplementary Fig. 2C, D). This indicates that drying method and flowering degree have a significant effect on VOCs of rose tea. Additionally, 24 compounds with rOAV and variable importance in projection (VIP) greater than 1 were selected (Supplementary Data 3c). Among them, trans-carveol, n-valeric acid cis-3-hexenyl ester, tetrahydro-4-methyl-2-(2-methyl-1-propenyl)-2H-pyran, and 2-phenylethyl ester acetic acid, were the main VOCs in the LTD rose samples. Hexanal, 2-pentyl-furan, (E)-2-hexenoic acid, and 2-hexanol played pivotal roles in VFD-RB. In addition, trans-β-ocimene, 2-undecanone, (Z)-3,7-dimethyl-1,3,6-octatriene, citral, and β-myrcene played pivotal roles in VFD-RC.

**Fig. 6:**
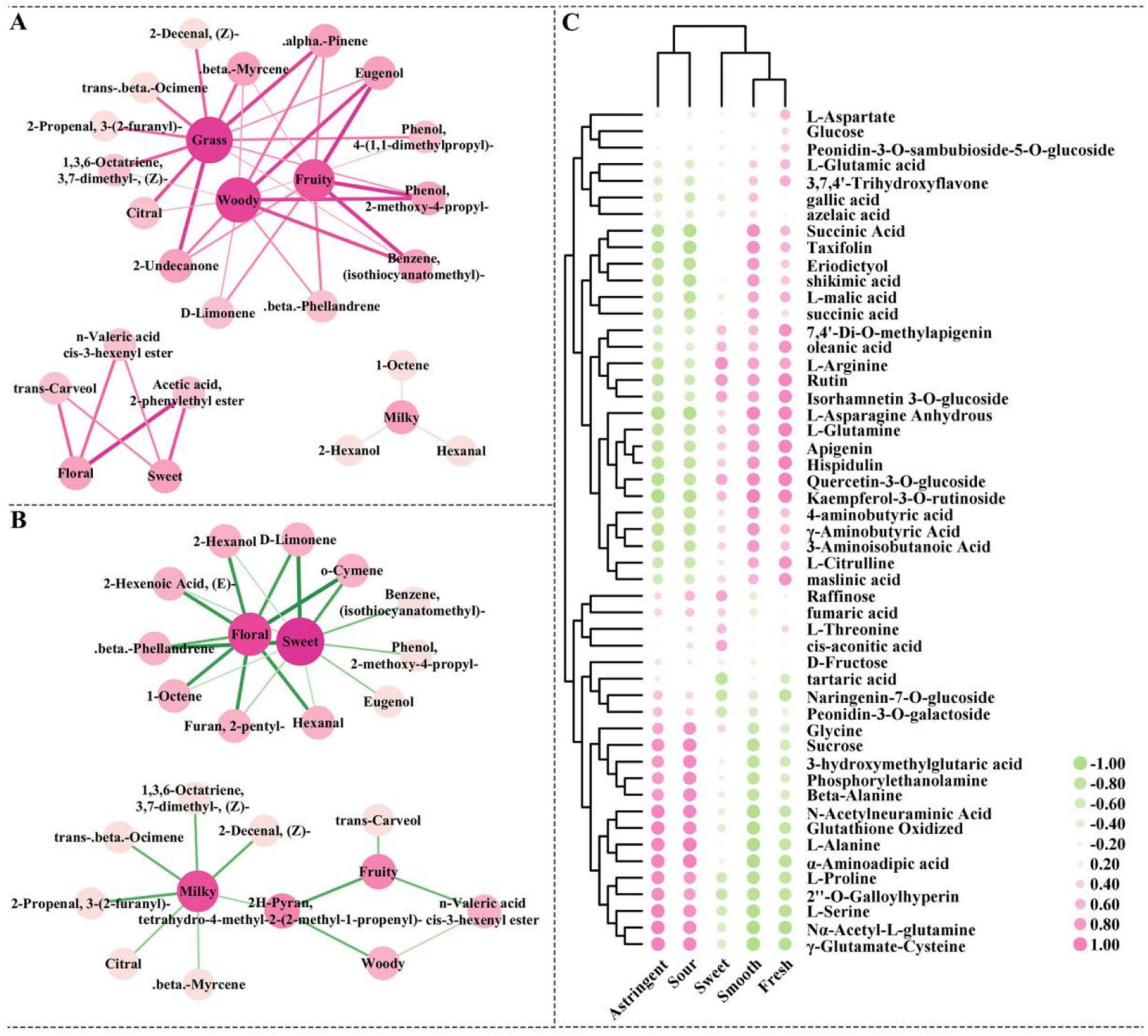
Network correlation analysis between key VOCs and nonvolatile compounds and aroma and taste of sensory quality analysis results. Correlation analysis between aroma metabolites (**A**, **B**) and taste metabolites (**C**) of rose tea and sensory quality analysis results. The red line indicates positive correlation (**A**) and the green line indicates negative correlation (**B**). The size of the nodes and the strength of their color indicate the degree of connectivity. The thickness of the line and the size of the circle indicate the strength of the correlation. The thicker the line and the larger the circle are, the stronger the correlation.

Potential link between taste metabolites and sensory characteristics was found using network analysis (Fig. 6A, B). The correlation analysis revealed a positively link between 1-octene, 2-hexanol, and hexanal and the milky aroma. Most of the milky aroma comes from lipid degradation pathways. For example, 2-hexanol is derived from the oxidation of linoleic acid, which is facilitated by lipoxygenase. Hexanal is considered one of the key aromas in milky-white tea^30^. The floral and sweet aromas were positively correlated with 2-phenylethyl acetate, cis-3-hexenyl n-valerate, and trans-carveol. Phenylethyl acetate has a rose like odor and is widely used to add fragrance or flavor to cosmetics and beverages^31^. Cis-3-hexenyl n-valerate is considered one of the characteristic aroma components of matcha^32^. The fruity and woody aromas were positively correlated with eugenol, 2-methoxy-4-propyl-phenol, and (isothiocyanatomethyl)-benzene. It has been established that eugenol is a crucial aroma component in rose-based products^33^. 2-Undecanone, α-pinene, β-myrcene, citral, (Z)-2-decenal, trans-β-ocimene and 3-(2-furanyl)-2-propenal had greater effects on the grass aroma. The majority of odorants that have a positive correlation with the aroma of grass have a negative correlation with the aroma of milky, and they are more concentrated in the VFD rose samples.

A similar situation occurs in the contribution of nonvolatile compounds to taste. Considering the significant differences in the contents of AAs, OAs, SSs, and flavonoids, conducting PLS-DA separately can provide a more comprehensive analysis of key nonvolatile compounds (Supplementary Fig. 3–6). Fifty-one compounds with a VIP greater than 1 were selected, including 21 amino acids, 12 organic acids, 4 SSs, and 14 flavonoids (Supplementary Data 4). Notably, for substances with strong positive correlation with astringent and sour, including γ-glutamate-cysteine, Nα-acetyl-L-glutamine, L-serine, 2”-O-galloylhyperin, L-proline, α-aminoadipic acid, L-alanine, glutathione oxidized, N-acetylneuraminic acid, β-alanine, phosphorylethanolamine, 3-hydroxymethylglutaric acid, glucose, glycine, tartaric acid, naringenin-7-O-glucoside, and peonidin-3-O-galactoside, had strong negative correlation with smooth and fresh. Additionally, L-arginine, raffinose, and L-threonine contributed more to the sweet aroma, while the most prevalent SSs in rose tea, glucose, and D-fructose, were negatively correlated with sweetness (Fig. 6C). Sensory studies have shown that sourness suppresses sweetness, and the inhibitory effect of sourness on sweetness is achieved by increasing the concentration of sourness^34^. In this study, the contribution of sucrose to sourness was much greater than that of sweetness, suggesting that a high concentration of sourness weakened the correlation between sucrose and sweetness.

## Discussion

This study utilized metabolomics and molecular sensory science to characterize the differences in metabolic composition and flavor of rose herbal tea with different forms and drying methods. One of the highlights of this article is the sensory evaluation of the popular rose herbal teas in the market. The distinct floral and sweet aroma of LTD roses can be attributed to the specific VOCs that are retained or generated during the LTD process^16^. This type of aroma is likely to be appealing to consumers who prefer a more traditional and delicate floral flavor in their tea. On the other hand, the more pronounced fruity and woody aroma of VFD roses may be a result of the chemical reactions that occur during the VFD process. These reactions could lead to the formation of new compounds or the transformation of existing ones, contributing to the unique flavor characteristics. The smoother and fresher taste of VFD rose infusion may also be related to the better preservation of certain nonvolatile compounds that affect the mouthfeel and overall taste experience^17^.

The metabolomics analysis provided valuable insights into the chemical composition of the rose teas. The identification of γ-aminobutyric acid, maslinic acid, glucose, Pn3G5G, and Cn3G5G as the most abundant nonvolatile metabolites suggests that these compounds play important roles in the taste and potential health benefits of rose herbal tea. For example, γ-aminobutyric acid has been associated with various biological functions, such as enhancing brain vitality, calming the mind, and lowering blood pressure^35^. The characteristic accumulation of nonvolatile compounds and VOCs in each type of rose tea indicates that the drying method and form of the rose herbal tea can have a significant impact on its chemical composition. This finding is consistent with previous research on the influence of processing methods on the quality of rose teas^13^. Similarly, the significant differences in soluble sugar content among different forms of rose tea, especially between rose buds and corollas at different stages of flower development, suggest that the selection of the rose form can also affect its taste and nutritional value.

Furthermore, PLS-DAs were identified 51 key nonvolatile and 24 volatile compounds, which is a valuable contribution to the understanding of the chemical basis of the flavor and quality of rose herbal tea. These key compounds can serve as markers for the identification and quality control of rose tea products. The correlation analysis further emphasized the relationship between these key compounds and the taste and aroma of the tea.

However, there are some limitations to this study. First, the study only focused on the metabolites and flavor composition of four common rose herbal tea products in the market. Further research could explore the evolution mechanism of key compounds in rose herbal tea during different processing stages. Second, the study did not investigate the stability of the identified key compounds over time. The shelf-life and storage conditions of rose tea can affect the quality and flavor of the product, and it is important to understand how the key compounds change during storage. Finally, the study did not conduct in-depth biological assays to determine the potential health benefits of the different rose teas. Future research could include in vitro and in vivo studies to evaluate the antioxidant, anti-inflammatory, and other biological activities of rose herbal tea.

In conclusion, this study has provided valuable insights into the differences in metabolic composition and flavor of rose herbal tea with different drying methods and forms. The findings can be used to guide the production and quality control of rose tea products, as well as to develop new and improved rose tea varieties. Further research is needed to address the limitations of this study and to explore the potential health benefits of rose herbal tea.

## Materials and methods

### Materials and reagents

Four rose herbal teas were made from three forms of roses, and processed using two drying methods, kindly provided by Shandong Huamei Biotechnology Co., Ltd. in Pingyin County (Jinan, Shandong Province, China). Representative appearance of rose herbal teas is presented in Fig. 1A. The three forms of roses were rose petal (RP, petals were picked from blooming flowers with visible stamens), rose buds (RB, conical flowers with petals that have not yet opened), rose corolla (RC, freshly opened flowers with petals that have not yet fully separated, invisible yellow stamens).

Methanol (MeOH), acetonitrile (ACN), ammonium acetate (NH(4)Ac), and formic acid (FA) were HPLC-grade (Tedia, Fairfield, OH, USA). The stock solutions were stored in MeOH. All of the water-soluble sugar standards were purchased from TCI (Shanghai), IsoReag (Shanghai), CNW (Shanghai Anpel), and Sigma-Aldrich (St. Louis, MO, USA). The extraction solution A of amino acid compounds was 70% methanol/water (v/v). The extraction solution B of SSs was methanol: isopropanol: water (3:3:2, v/v/v).

### Drying processes

The two drying processes used were LTD and vacuum freeze drying (VFD). LTD was achieved through a low-temperature heat pump dehumidification process. Fresh rose flowers (RP and RC) were selected and sorted, and then placed on a tray before being pushed into a drying room, with a drying temperature range of 35–60 °C. The processing time was set as 20–22 h. By using a dehumidification heat pump to gradually condense and recover moisture, the rose completes the drying process.

The freeze-drying process used ultra-low temperature VFD equipment for processing. Fresh rose flowers (RB and RC) were selected and sorted, placed on a tray, and then pushed into a quick freezing tunnel. The roses were frozen at −41 °C for at least 3 h. Then push it into the vacuum chamber. Subsequently, the sample was vacuum freeze-dried under the following conditions: a vacuum degree below 80 Pa, a cold hydrazine temperature below −30 °C, and a heating plate temperature below 40 °C, for 20 h. Water vapor molecules migrate and adsorb from frozen flowers to cold hydrazine, and roses slowly complete the drying process.

The LTD-RBs have significant quality damage, and the VFD-RPs were fragile and difficult to maintain in their intact state. Therefore, the LTD-RB and VFD-RP were removed from the material selection. Three consecutive batches of samples were combined into a mixed sample for testing, with three replicates for each sample.

### Sensory evaluation

All the tea tasters who participated in the examination provide informed consent before the sensory examination began. Sensory evaluation was conducted by six trained evaluators (2 males and 4 females, aged 30–35 years) in conformity to the national standard “Tea Sensory Evaluation Procedure” (GB/T 23776-2018). The rose herbal tea was brewed with water (95–100 °C) at 1:100 for 5 min. The sensory qualities of rose tea were assessed by quantitative descriptive analysis with a 10-point hedonic scale (0 indicates no intensity or undetectable intensity. 3 is weak, 5 is moderate, 7 is high, and 10 is extremely high.)^36^. The intensity increases with a higher score. The sensory attributes, including the floral, sweet, fruity, milk, woody, and grass aroma attributes, as well as the sour, astringent, fresh, smooth, and sweet taste attributes, were evaluated. The scores of sensory evaluation was listed in Supplementary Data 1.

### Quantitative analysis of amino acids (AAs) in rose herbal teas

Following the material’s thawing and crushing, 0.05 g of the sample powder and 0.5 mL of extraction solution A were combined and vortexed for 3 min. The mixture was then centrifuged at 12,000 rpm at 10 °C for 10 min. Subsequently, the supernatant was moved and refrigerated for 30 min at −20 °C. Finally, the supernatant was thawed and centrifuged once more for 10 min at 10 °C at 12,000 rpm for LC-ESI-MS/MS analysis.

An ACQUITY BEH Amide (i.d. 2.1 × 100 mm, 1.7 µm) column was used to qualitatively analyze the amino acids, comprised solution A (water with 2 mM NH(4)Ac and 0.04% FA), and solution B (ACN with 2 mM NH(4)Ac and 0.04% FA). The injection volume was 2 µL, the temperature was 40 °C, and the flow rate was 0.4 mL/min. The gradient in the solvent system was started at 90% B (0 to 1.2 min), 60% B (9 min), 40% B (10 to 11 min), and 90% B (11.01 to 15 min).

### Quantitative analysis of organic acids (OAs)

The sample preparation and extraction were the same as those for the AAs. An ACQUITY HSS T3 column (2.1 × 100 mm, 1.8 μm) was used with a mobile phase comprising 0.05% FA aqueous (solution A), and ACN with 0.05% FA (solution B). The injection volume and the temperature were the same as those for the AAs, the flow rate was 0.35 mL/min. The gradient of B was set at 5% at 0 min, increased to 95% at 8 to 9.5 min, and then ramped back to 5% at 9.6 to 12 min.

### Quantitative analysis of water-soluble sugars (SSs)

A total of 0.02 g of the sample powder and 0.5 mL of extraction solution B were combined and vortexed for 3 min followed by ultrasonicated for 30 min. The mixture was then centrifuged at 12,000 rpm at 10 °C for 5 min. A mixture of 20 μL internal standard (1000 μg/mL) and 50 μL of the supernatant was evaporated and subjected to freeze-drying for further derivatize. A methoxyamine hydrochloride in pyridine solution (100 μL, 15 mg/mL) was added into the residue and then incubated for 2 h at 37 °C. Subsequently, the mixture was added with BSTFA (100 μL) and then vortexed and maintained at 37 °C for 30 min. The combination was diluted to an appropriate concentration for further analysis.

An Agilent 7890B-7000D (GC-MS) system was utilized with a DB-5MS column. The injection volume was 2 μL with a split ratio of 3:1. The flow rate of the carrier gas (helium) was 1 mL/min. The transfer line and ion source temperatures were 280 °C and 230 °C, respectively. The oven temperature was set to 150 °C for 1 min, then 200 °C at 5 °C/min, 300 °C at 16 °C/min, 320 °C at 20 °C/min, and kept at 320 °C for 5.5 min.

### Quantitative analysis of flavonoids

The sample powder (20 mg) was extracted with 0.5 mL of extraction solution A. For quantification, the internal standard (IS) (4000 nmol/L, 10 μL) was added. The mixture was centrifuged at 12,000 rpm for 5 min at 10 °C after 30 min of sonication. For further UPLC-ESI-MS/MS, the supernatant was filtered using a 0.22 μm membrane filter.

The Waters ACQUITY UPLC HSS T3 C18 column (100 mm × 2.1 mm × 1.8 µm) was used with 0.05% FA aqueous (solution A), and ACN with 0.05% FA (solution B). The injection volume, the temperature, and the flow rate were the same as those for the OAs. The gradient in the solvent system was started at 0 to 1 min, 10–20% B; 1–9 min, 20–70% B; 9–12.5 min, 70–95% B; 12.5–13.5 min, 95% B; 13.5–13.6 min, 95–10% B; and 13.6–15 min, 10% B.

### Volatilomics analysis

HS-SPME and GC-MS were combined to investigate the variations in aromatic components and their concentrations of rose tea. Five hundred milligrams (1 mL) of powder was rapidly transferred to a head-space injection vial containing 20 ml of saturated NaCl. When conducting SPME analysis, the vial was first preheated at 60 °C for 5 min, and then a 120 µm DVB/CWR/PDMS fiber was exposed in the headspace of the sample for 15 min, while maintaining the temperature at 60 °C.

The VOCs were desorbed for 5 min in a splitless mode at 250 °C at the injection port of the GC apparatus. To identify and quantify VOCs, an Agilent 8890-7000D (GC-MS) system was utilized with a DB-5MS column (5% phenyl-polymethylsiloxane). The detector temperature was maintained at 280 °C, and the injector temperature was maintained at 250 °C. The flow rate of the carrier gas (helium) was 1.2 mL/min. The transfer line was set at 280 °C, the ion source was set at 230 °C, and the quadrupole mass detector temperatures was set at 150 °C. The oven temperature was set at 40 °C(3.5 min), raised 100 °C at 10 °C/min, 180 °C at 7 °C/min, 280 °C at 25 °C/min, and maintained for 5 min at 280 °C.

### Multivariate analysis

The original data were listed in Supplementary Data. Hierarchical cluster analysis (HCA) was carried out using TBtools software. PCA was applied to investigate the metabolome variations among those rose herbal tea by using Simca 14.0. The PLS-DA model was validated through 200 permutation experiments. To centralize and standardize the identified differentially abundant metabolite contents, TCA was applied using online platforms (https://www.omicshare.com/tools). One-way ANOVA (LSD test) was used in IBM SPSS to calculate significant differences between different rose samples. The Spearman correlation coefficient between the metabolite content and sensory properties of all samples was calculated. Simca 14.0 and Cytoscape (version 3.10.2) were utilized to visualize the relationships between flavor metabolites and the sensory evaluation (*p* < 0.05 and |r| > 0.6).

## Supplementary information

Supplementary Information

Supplementary Data 4

## Data Availability

Data is provided within the manuscript or supplementary information files.
