# Supplementary material for: Metabolomics and sensory evaluation combined analysis reveal the effect of processing methods and different forms on the flavor of rose herbal tea
Source: NPJ Sci Food. 2025 Mar 29;9:45. doi: 10.1038/s41538-025-00387-x (PMC11954921; doi:10.1038/s41538-025-00387-x)
Supplement: Supplementary file 1 — Supplementary Information [file 41538_2025_387_MOESM1_ESM.pdf]

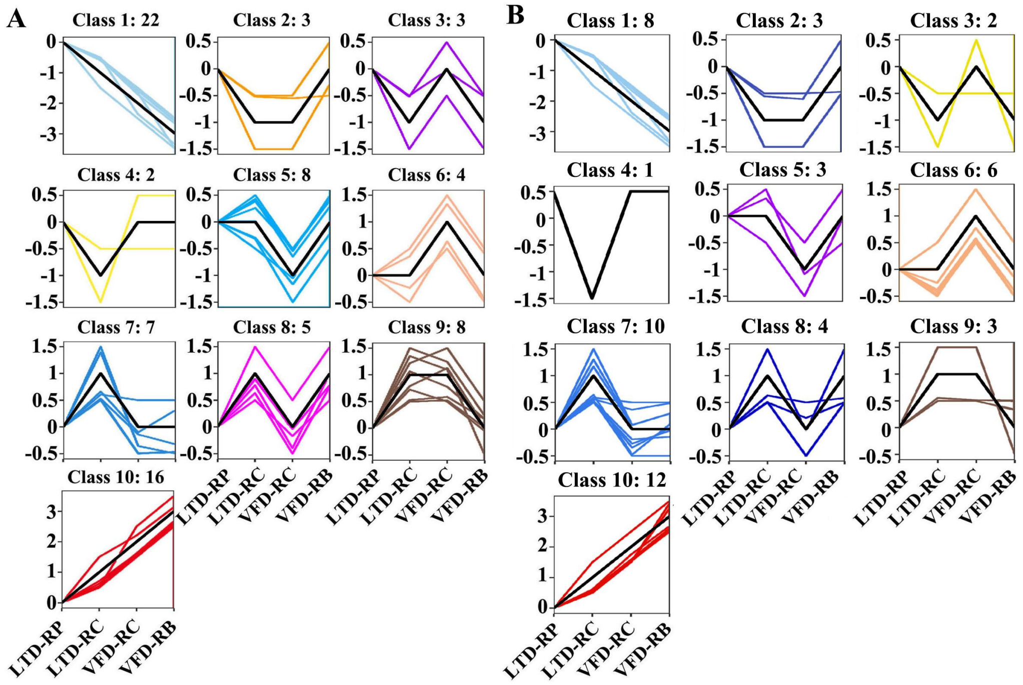

Supplementary Figure 1. Trend clustering analysis of AAs and OAs accumulation patterns of the four types of rose herbal teas.

A, Trend clustering analysis of amino acids accumulation patterns among the four types of rose herbal teas. B, Trend clustering analysis of organic acids accumulation patterns among the four types of rose herbal teas.

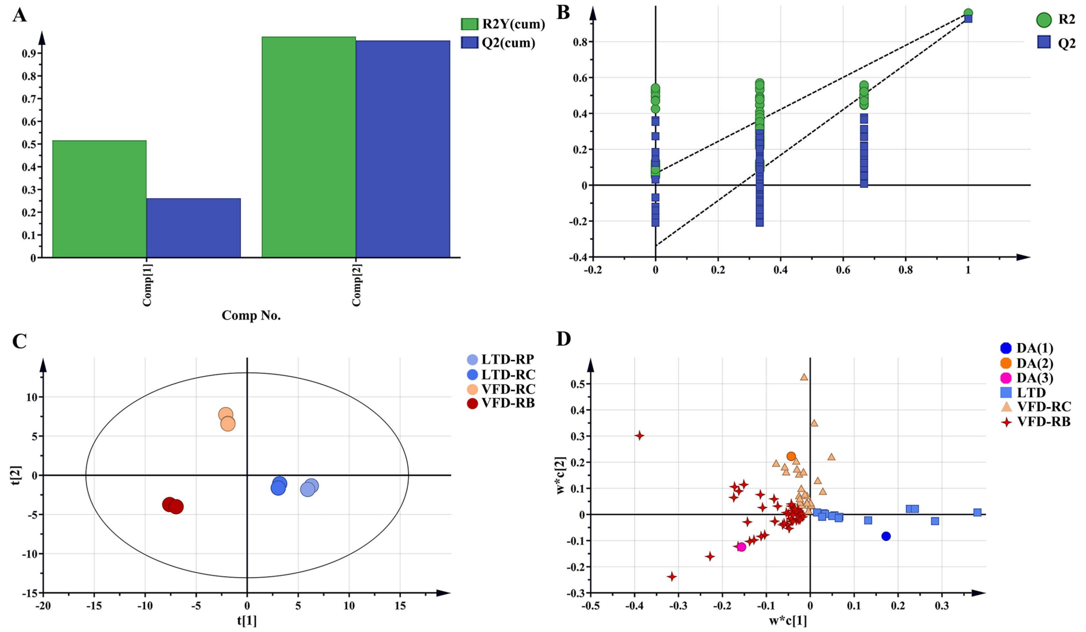

Supplementary Figure 2. The Partial Least Squares Discriminant Analysis (PLS-DA) of VOCs that directly contribute to the flavor of the four types of rose herbal teas samples (rOAVs≧1).

A, Summary of fit plot of PLS-DA based on cross-validation. B, Validate model with 200 permutation tests. C, The PLS-DA score scatters plot based on t1 versus t2 (R2X = 0.912, R2Y = 0.973, Q2 = 0.956). D, PLS-DA loading scatter plot based on p1 versus p2.

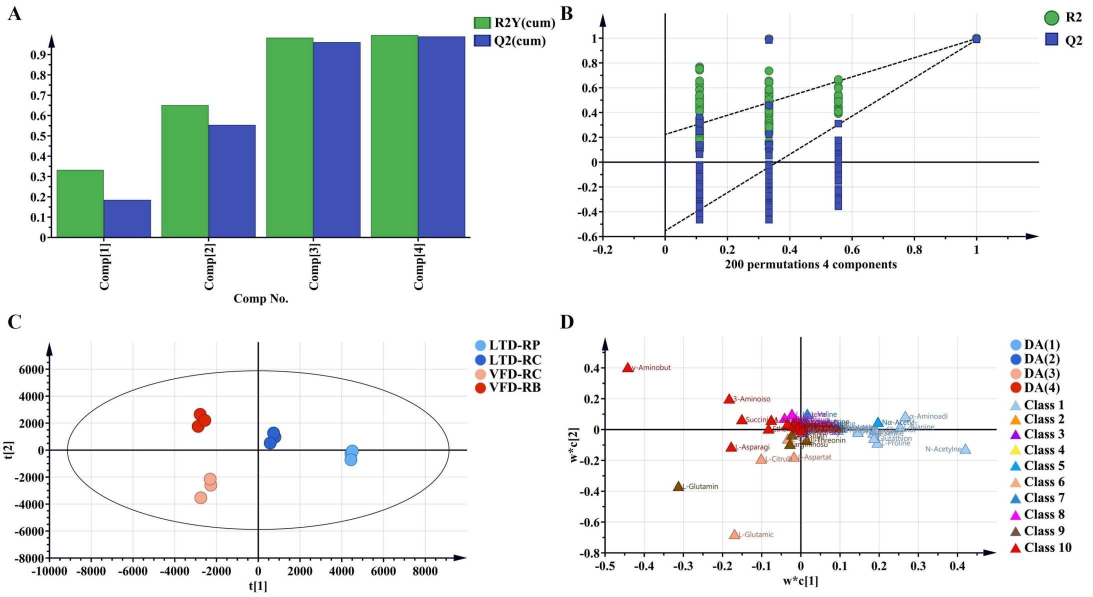

Supplementary Figure 3. The PLS-DA of AAs of the four types of rose herbal teas samples. A, Summary of fit plot of PLS-DA based on cross-validation. B, Validate model with 200 permutation tests. C, The PLS-DA score scatters plot of AAs based on t1 versus t2 ($R2X = 0.993$, $R2Y = 0.996$, $Q2 = 0.989$). D, PLS-DA loading scatter plot based on p1 versus p2.

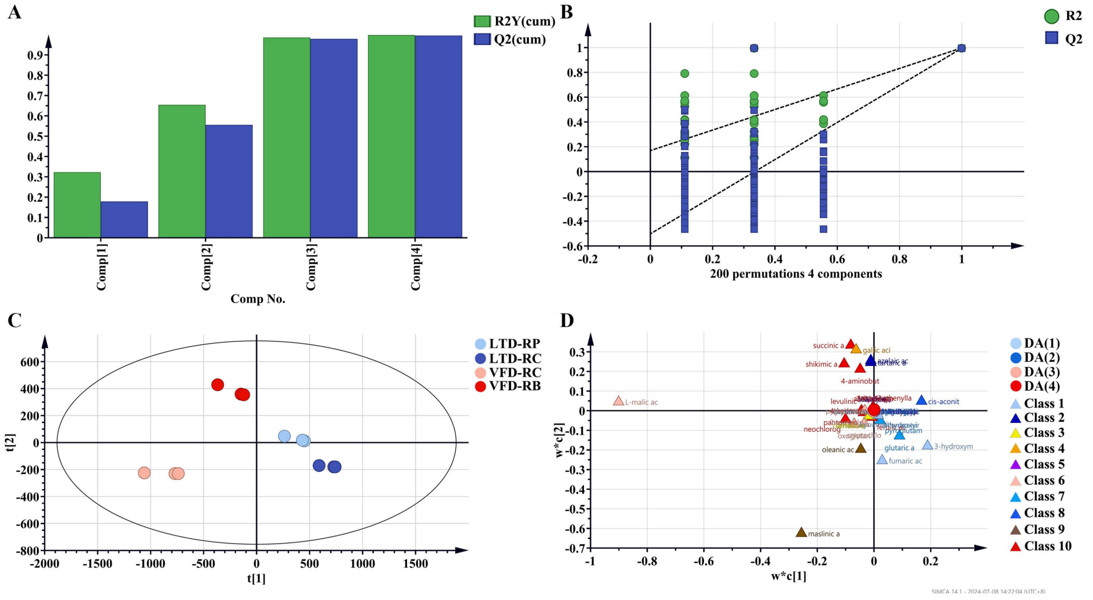

Supplementary Figure 4. The PLS-DA of OAs of the four types of rose herbal teas samples. A, Summary of fit plot of PLS-DA based on cross-validation. B, Validate model with 200 permutation tests. C, The PLS-DA score scatters plot of OAs based on t1 versus t2 ($R2X = 1$, $R2Y = 0.998$, $Q2 = 0.995$). D, PLS-DA loading scatter plot based on p1 versus p2.

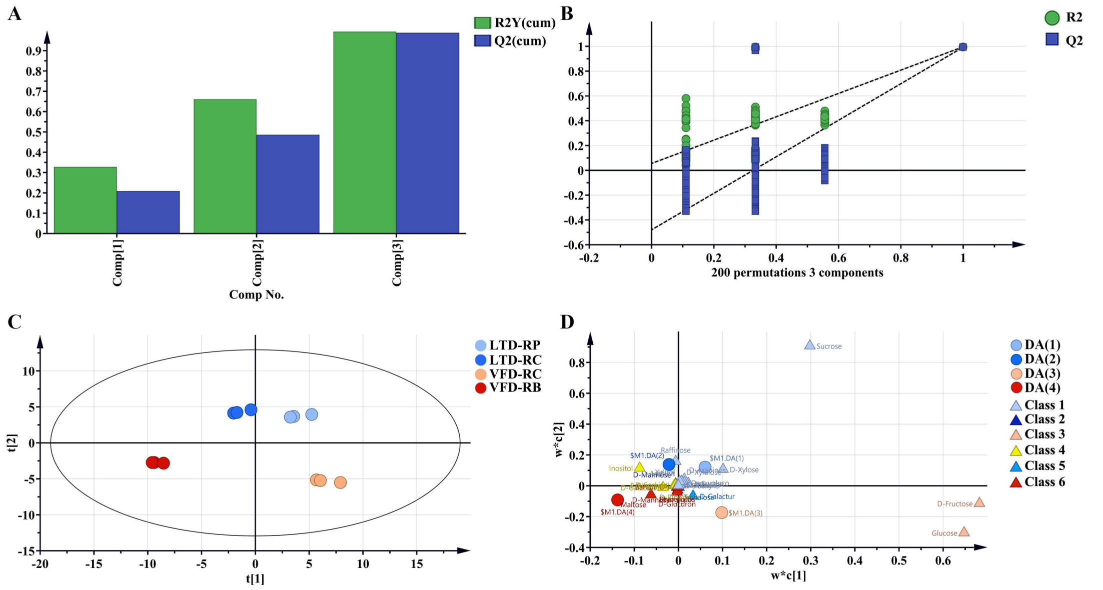

Supplementary Figure 5. The PLS-DA of SSs of the four types of rose herbal teas samples.

A, Summary of fit plot of PLS-DA based on cross-validation. B, Validate model with 200 permutation tests. C, The PLS-DA score scatters plot of SSs based on t1 versus t2 (R2X = 0.998, R2Y = 0.994, Q2 = 0.988). D, PLS-DA loading scatter plot based on p1 versus p2.

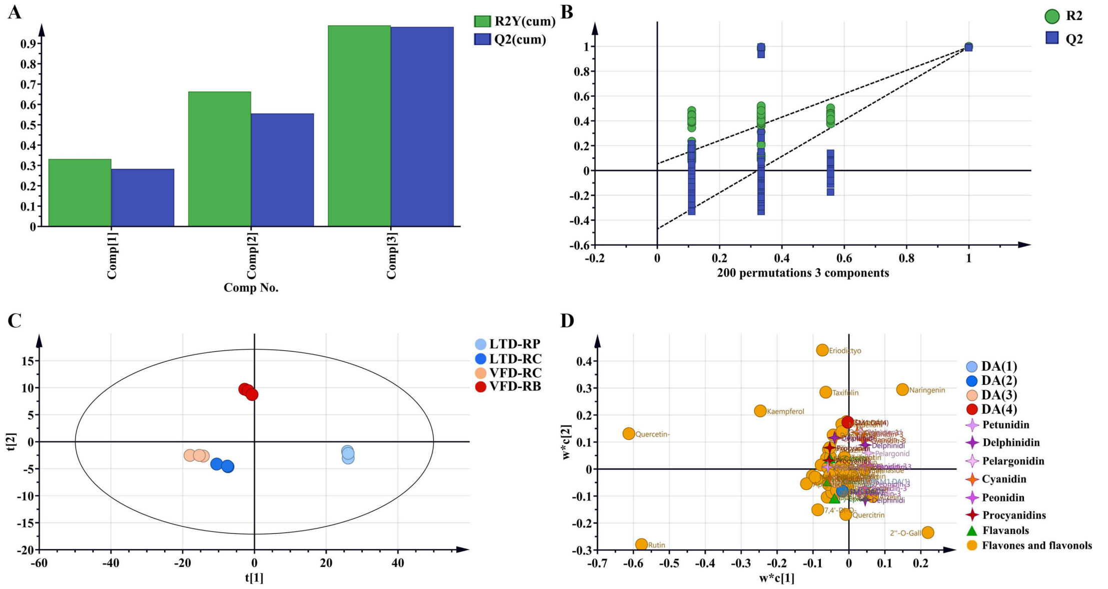

Supplementary Figure 6. The PLS-DA of flavonoids of the four types of rose herbal teas samples.

A, Summary of fit plot of PLS-DA based on cross-validation. B, Validate model with 200 permutation tests. C, The PLS-DA score scatters plot of flavonoids based on t1 versus t2 (R2X = 0.994, R2Y = 0.989, Q2 = 0.981). D, PLS-DA loading scatter plot based on p1 versus p2.

Supplementary Data

Supplementary Data 1. The quantitative descriptive analysis scores of taste and aroma.
Supplementary Data 2a. Amino acids and their derivatives in four rose samples were detected using LC-MS/MS.
Supplementary Data 2b. Organic acids of four rose samples were detected using LC-MS/MS.
Supplementary Data 2c. Soluble sugars in four rose samples were detected using GC-MS.
Supplementary Data 2d. Flavonoids in four rose samples were detected using LC-MS/MS.
Supplementary Data 3a. The concentrations and odor activity values of VOCs based on GC-MS and rOAVs (rOAV ≥ 1) in four rose herbal teas.
Supplementary Data 3b. The classification of VOCs based on PCA loading scatter plot in four rose herbal teas.
Supplementary Data 3c. The odor activity values and concentrations of key VOCs with VIP>1 in PLS-DA analysis of four rose herbal teas.
Supplementary Data 4. Key nonvolatile compounds with VIP>1 in PLS-DA analysis of four rose herbal teas.
